# Scar Endometriosis vs Hemangioma: A Diagnostic Conundrum

**DOI:** 10.7759/cureus.47674

**Published:** 2023-10-25

**Authors:** Hrithik Dakssesh Putta Nagarajan, Keerthivasan Selvanathan, Vrijesh Gopalakrishnan, Ram Vivek Ramamoorthy, Jeyachitra Gopalakrishnan

**Affiliations:** 1 Department of Dermatology, Madurai Medical College, Madurai, IND; 2 Department of General Surgery, Madurai Medical College, Madurai, IND; 3 Department of Obstetrics and Gynaecology, Nithilaa Nursing Home, Madurai, IND

**Keywords:** catamenial changes, skin, case report, excision and biopsy, cyclical abdominal pain, menstrual cycle, hemangioma, scar site endometriosis

## Abstract

Scar endometriosis refers to the presence of endometrial glands and stroma at the site of a scar. Hemangiomas, on the other hand, are benign vascular tumors. In this case report, we unravel the clinical enigma around a patient who presented with a painful mass at the previous cesarean section scar site. Initially, we were confident that this was ectopic endometrium presenting as scar endometriosis. However, our journey took an unexpected turn when histopathological findings contradicted our clinical suspicions. Here, we delve into the intricate details of this captivating case, shedding light on the complexities of the diagnosis we faced.

## Introduction

Endometriosis is a gynecological disease, characterized by the development and presence of endometrial tissue in places outside of the uterine cavity. The most commonly affected areas include the ovaries (54.9%), followed by the posterior broad ligament (35.2%), anterior cul-de-sac (34.6%), posterior cul-de-sac (34.0%), and the uterosacral ligament (28.0%) [1]. Scar endometriosis is a relatively rare form of this condition, with a reported incidence of 0.08% among women who have had cesarean sections [2]. It typically occurs in the anterior abdominal wall, at the site of previous cesarean section scars.

Hemangiomas are benign overgrowths of blood vessels and endothelial cells, found either in the outer layers of the skin, deeper layers of the skin, internal organs, subcutaneous tissue, or sometimes in multiple locations simultaneously. Hemangiomas are categorized as capillary or cavernous based on the size of the vascular channels. Capillary hemangiomas have small diameter vascular channels. On the other hand, cavernous hemangiomas have large diameter vascular channels [3]. While hemangiomas are commonly seen in infants, encountering them in adults, especially within post-cesarean section scars, is exceptionally rare. Many cases of hemangioma occur spontaneously without any identifiable cause, while others are associated with factors such as injuries, burns, immobilization using casts, pregnancy, and the use of antiretroviral therapy [4].

## Case presentation

**Figure 1:**
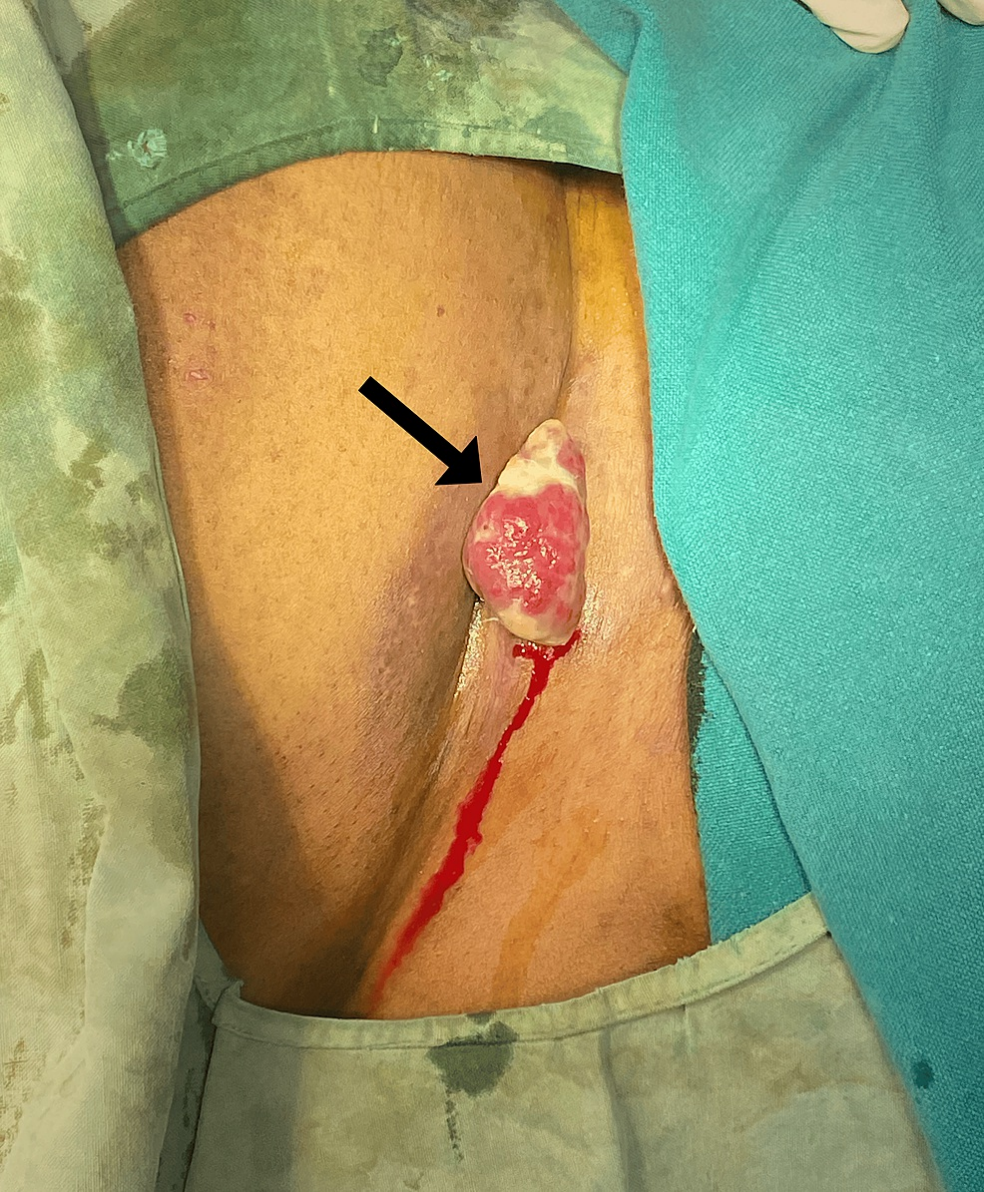
Image showing the presence of mass (arrow) in the lateral aspect of the previous LSCS scar on the right side of the abdomen. LSCS: lower segment cesarean section.

A 34-year-old South Asian female presented to the clinic with chief complaints of swelling and associated pain in the right lower abdomen, specifically over the lateral aspect of the previous cesarean section scar on the right side, which had been bothering her for the past 10 months. The patient has two healthy children delivered via lower segment cesarean section (LSCS). The patient noticed the development of this swelling during her second pregnancy, around the fifth month, over the LSCS scar of the first pregnancy (Figure 1). Initially, it was suspected to be a keloid.

The patient sought medical attention five months after the delivery of the second child, due to the emergence of pain in the swelling, coinciding with the resumption of her menstruation, which occurred three months postpartum. During the initial three months following delivery, the patient did not experience any menstrual flow due to lactational amenorrhea.

The size of the swelling was noted to be equivalent to that of a soybean (0.7 cm x 0.4 cm) by the operating surgeon during the delivery of the second baby. But then, it gradually increased in size to measure 3 cm x 2 cm. Throughout the second pregnancy and the initial three months following delivery, the skin over the swelling was normal. However, with the onset of menstruation, the skin over the swelling began to peel off and was accompanied by serous discharge. The swelling also exhibited color changes in relation to the menstrual cycle. It appeared with a bright reddish hue just before the onset of menses and turned pale pinkish after menstruation. It is important to note that the patient did not complain of any bleeding from the swelling.

Additionally, the patient noticed that the swelling became harder in consistency during menstruation, compared to its firm state at other times. She complained that the intensity of the pain varied in accord with her menstrual cycle. The pain typically spiked during the time of menstrual blood flow, which usually lasts for about five days in this patient. However, she also reported a period of gradual waxing of pain, for about three days before the onset of menstrual flow, and a subsequent period of waning of pain, for about three days after the passage of menses. The patient also reported experiencing intermittent pricking pain, that lasts for about five minutes per episode, over the swelling at times other than during menstruation.

Investigations

Ultrasound Imaging

After the initial clinical workup was done, she was sent for an ultrasound imaging of the mass. The scan showed a heteroechoic lesion, measuring 2.9 cm x 1.8 cm at the scar site in the subcutaneous plane. Color flow revealed rich vascularity.

Magnetic Resonance Imaging

**Figure 2:**
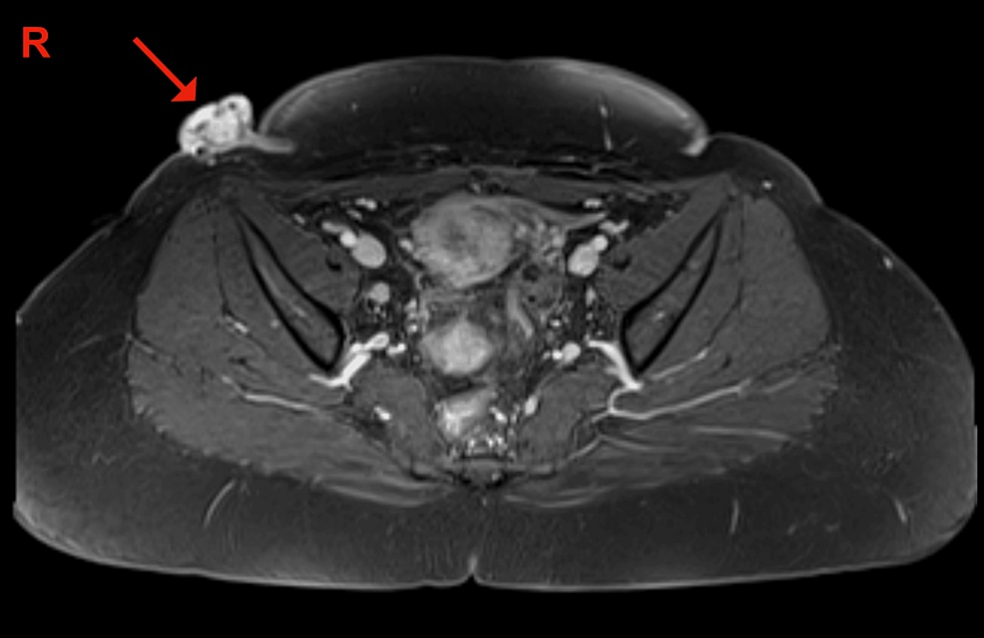
Magnetic resonance imaging (MRI) of the abdomen and pelvis revealing the mass (arrow). It is seen as an intensely enhancing soft tissue mass.

Further imaging studies were considered necessary. So a magnetic resonance imaging (MRI) of the abdomen and pelvis was taken. It showed a well-defined polypoidal, intensely enhancing soft tissue mass with internal hemorrhagic foci and significant vascularity, in the lateral aspect of the postoperative scar site on the right side measuring 3.2 cm x 2.2 cm x 1.8 cm in size, with adjacent skin thickening (Figure 2).

Treatment

**Figure 3:**
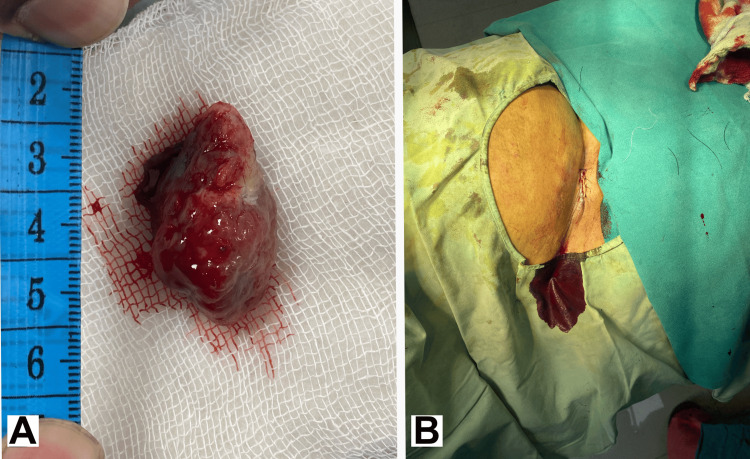
Image of the excised mass and postoperative image. A) Image of the excised mass with a centimeter (cm) scale for reference. B) Postoperative image showing the incision site closed with polypropylene sutures.

After a proper workup, excision and biopsy of the swelling was planned. Owing to the easy excisability of the lesion, no trial of hormonal drug therapy was tried. The excision and biopsy was performed under local anesthesia and was uneventful without any complications. The excised mass (Figure 3A) was sent for histopathology. The incision site was then closed with intermittent polypropylene sutures (Figure 3B). The patient was discharged, with advice to return in case of persistence of symptoms, and to return to the clinic after two weeks for the removal of sutures. Nonsteroidal anti-inflammatory drugs (NSAIDs) were prescribed for three days following surgery for the relief of postoperative pain.

Histopathology

**Figure 4:**
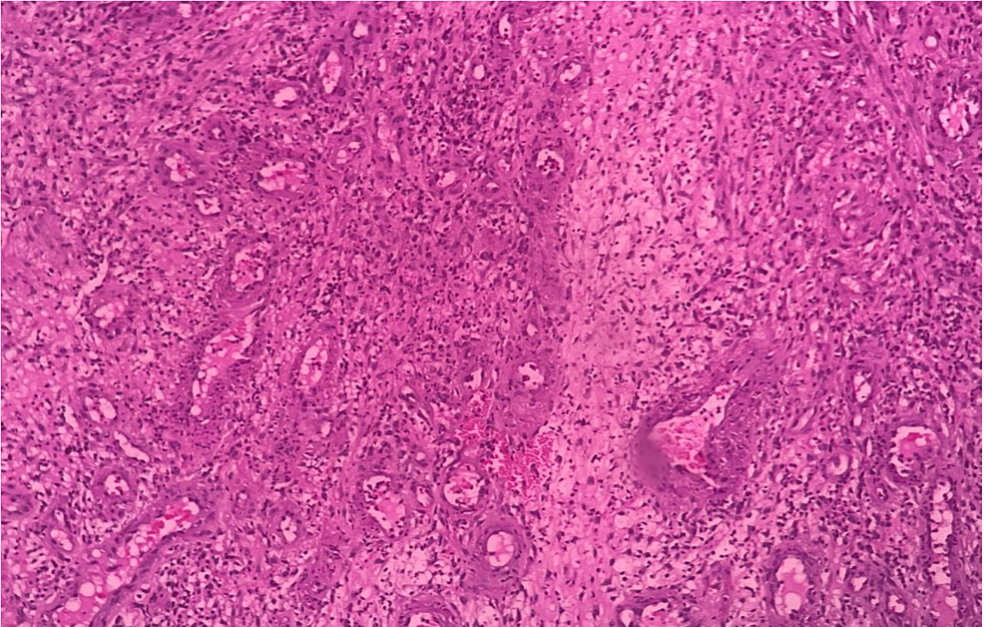
Histopathology of the excised mass showing fibrocollagenous tissue enclosing numerous proliferating blood vessels lined by prominent endothelial cells surrounded by neutrophils and lymphocytes.

The excised mass was sent in for histopathological studies. The cut surface of the mass was noted to be gray-brown in color. A thorough study of sections from the sample, under the microscope, showed fibrocollagenous tissue with dilated proliferating blood vessels, areas of hemorrhage and inflammatory cell infiltrates (Figure 4). Endometrial glands and stroma were nowhere to be found, and the other features were suggestive of a capillary hemangioma. So, a definitive diagnosis of hemangioma was made.

Follow-Up

After two weeks, the patient reported back to the clinic for removal of sutures. The scar site was healthy with no discharge. No residual signs were noted, and the sutures were removed. Telephonic follow-up after one month from the date of the procedure was done. The patient was informed of no residual symptoms.

## Discussion

Scar endometriosis typically presents with localized cyclical pain, often occurring in conjunction with menstruation. The pain is characterized as sharp, localized discomfort that intensifies during menstruation, reflecting the influence of hormones on endometrial tissue. Other manifestations include a palpable mass or swelling at the cesarean scar site which may present with tenderness [5,6]. The elicited history of cesarean section and correlation of symptoms with the menstrual cycle were pivotal in our case and led to a provisional diagnosis of scar endometriosis.

The clinical presentation of hemangioma depends on its location and depth within the body. The majority present as a single localized cutaneous mass. Hemangiomas most commonly occur on the head and neck region, accounting for 60% of cases. Following that, 25% of the cases are seen on the trunk, as in this case. Although 25% is a significant share, all hemangiomas are commonly observed only in infants and are rare to be seen in adults. Hemangiomas can be superficial, deep, or mixed with components of both superficial and deep layers. Superficial lesions involve the superficial dermis and are raised, lobulated, and bright red. Deep hemangiomas, also called subcutaneous hemangiomas, arise from the reticular dermis and/or the subcutis layer and appear as bluish-hued nodule or plaque. Mixed hemangiomas present with features of both locations [7]. The presentation of hemangioma tends to be consistent and unaffected by the menstrual cycle.

Hemangiomas may sometimes present with pain. However, it is exceedingly rare for superficial hemangiomas to be painful, especially when the surface is intact. Furthermore, it is even rarer for the pain to follow a cyclical pattern. Only hemangiomas with ulceration and spinal hemangiomas are commonly known to cause pain. There is very little literature providing evidence for hemangiomas presenting with such cyclical features. In the case report by Ortiz-Rey et al. [8], evidence for catamenial changes with the color, size, and intensity of pain of a similar lesion is given. They also faced the same dilemma as we faced and initially thought it was a case of cutaneous endometriosis and had the lesion excised. In that case report, details of a hemangioma on the lower abdomen of a woman taking oral contraceptives are given. In that patient, the catamenial nature of these changes was precisely elicited, as the menstrual cycle was under control with the use of monophasic contraceptive pills [8].

Although there are a few more similar clinical evidence in medical literature [9-12], to our knowledge, this is the first case in which this type of lesion presented over the scar from previous LSCS in a post-cesarean section female, causing utmost confusion in the diagnosis as these features were very typical of a case of scar endometriosis. These clinical pieces of evidence, in the form of case reports, support the possibility of sex steroid influence on vascular lesions. In most of these cases, the results of immunohistochemistry for estrogen and progesterone receptors came back negative. But estrogen by itself, without acting on the receptors, can cause venodilation leading to changes in the lesion as observed in our patient [13]. So the catamenial fluctuations in the levels of estrogen may be responsible for the catamenial changes in these vascular lesions.

## Conclusions

Our case highlights the diagnostic challenges we faced in differentiating scar endometriosis from hemangioma. While scar endometriosis was our initial suspicion due to its clinical presentation, histopathological examination of the excised tissue was crucial for confirming the diagnosis as hemangioma. Although rare, it is vital to keep hemangioma as a probable differential in our minds when faced with similar clinical situations. The importance of accurate diagnosis in cases like these cannot be emphasized enough. Misdiagnosis can lead to inappropriate treatments, such as hormonal therapies commonly used for endometriosis, being administered for cases of hemangiomas, which would be ineffective in treating them. In contrast, a precise diagnosis enables the implementation of the most appropriate treatment plan. Further research is needed to better understand the clinical and histological characteristics of these conditions, facilitating early and accurate diagnosis and improving patient outcomes.
